# Ethical and human rights considerations in public health in low and middle-income countries: an assessment using the case of Uganda’s responses to COVID-19 pandemic

**DOI:** 10.1186/s12910-020-00523-0

**Published:** 2020-09-22

**Authors:** John Barugahare, Fredrick Nelson Nakwagala, Erisa Mwaka Sabakaki, Joseph Ochieng, Nelson K Sewankambo

**Affiliations:** 1 Department of Philosophy, College of Humanities and Social Sciences, Makerere University, P. O. Box 7062, Kampala, Uganda; 2 Department of Internal medicine, Mulago National Referral Hospital, P. O. Box 7051, Kampala, Uganda; 3 Department of Anatomy, School of Biomedical Sciences, College of Health Sciences, Makerere University, P. O. Box 7062, Kampala, Uganda; 4 Department of Medicine, School of Medicine, Makerere University College of Health Sciences, P. O. Box 7062, Kampala, Uganda

**Keywords:** Public health, Human rights, Ethics, COVID-19, Low income countries, Public health emergencies

## Abstract

**Background:**

In response to COVID-19 pandemic, the Government of Uganda adopted public health measures to contain its spread in the country. Some of the *initial* measures included refusal to repatriate citizens studying in China, mandatory institutional quarantine, and social distancing. Despite being a public health emergency, the measures adopted deserve critical appraisal using an ethics and human rights approach. The goal of this paper is to formulate an ethics and human rights criteria for evaluating public health measures and use it to reflect on the ethical propriety of those adopted by the government of Uganda to contain the spread of COVID-19.

**Main body:**

We begin by illustrating the value of ethics and human rights considerations for public health measures including during emergencies. We then summarize Uganda’s social and economic circumstances and some of the measures adopted to contain the spread of COVID-19. After reviewing some of the ethics and human rights considerations for public health, we reflect upon the ethical propriety of some of Uganda’s responses to COVID-19. We use content analysis to identify the measures adopted by the government of Uganda to contain the spread of COVID-19, the ethics and human rights considerations commonly recommended for public health responses and their importance. Our study found that some of the measures adopted violate ethics and human rights principles. We argue that even though some human rights can sometimes be *legitimately* derogated and limited to meet public health goals during public health emergencies, measures that infringe on human rights should satisfy certain ethics and human rights criteria. Some of these criteria include being *effective, strictly necessary, proportionate* to the magnitude of the threat, *reasonable* in the circumstances*, equitable*, and *least restrictive*. We reflect on Uganda’s initial measures to combat the spread of COVID-19 and argue that many of them fell short of these criteria, and potentially limit their effectiveness.

**Conclusion:**

The ethical legitimacy of public health measures is valuable in itself and for enhancing effectiveness of the measures. Such legitimacy depends on the extent to which they conform to ethics and human rights principles recommended for public health measures.

## Background

On December 31, 2019, China reported 44 cases of “Pneumonia of unknown cause” to the World Health Organization (WHO) [1]. On January 30, 2020, this disease that later came to be known as coronavirus disease 2019 (COVID-19) was declared “a Public Health Emergency of International Concern (PHEIC)” [2]. By July 6, 2020, the global prevalence of COVID-19 infections had soared to over eleven million cases with over half a million deaths; in Africa the number of infections was 369,928 while 6974 had died [3]. Around the same time, Uganda had registered 953 cases of infection, 892 recoveries and no death [4]. One of the worrying characteristics of COVID-19 is its rapid rate of spreading both geographically and in terms of cases [5, 6]. In the wake of this pandemic, the Government of Uganda adopted several preventive measures to contain its spread, mainly ensuring physical and social distancing. Some of the *initial* measures included partial lockdown of business and social activities - closure of all education institutions, suspension of all communal worship, and political gatherings, among others. Some of the initial measures also included recommended, and in some cases, mandated hand washing; denying students studying in China to return home; voluntary self-quarantine; mandatory institutional quarantine at one’s own cost; suspension of both public and private transport; and imprisonment for non-compliance with the measures [7–9].

Even though these measures are potentially very effective at reducing human-to-human infections, some of them present latent ethical and human rights controversies, despite the general legitimacy of limiting and derogating human rights during public health emergencies (PHEs). Such legitimacy is partly derived from John Stuart Mill’s ‘Harm Principle’ [10]; the Siracusa Principles, particularly Clause 25 [11]; and Uganda’s Public Health Act, 1935 [12]. According to these sources, governments can justifiably limit the exercise of individual liberties and freedoms, such as freedom of movement and association or the right to privacy, especially if such exercise is deemed likely to cause a public health harm in the form of spreading infectious diseases or causing injuries. Although ethics and human rights are sometimes treated as separate fields, in the context of public health, they largely overlap. Most of the ethical controversies about public health measures arise from the manner and extent to which such measures impact people’s rights and freedoms. Consequently, certain ethical and human rights considerations should guide such limitations. For this reason, in addition to declaring COVID-19 a PHEIC, the WHO Director-General advised countries to strike a balance between protecting health, minimizing economic and social disruption, and *respecting human rights* [13, 6] (emphasis added). Although the WHO has previously made efforts to encourage governments to ensure ethical preparedness by developing ethical frameworks for public health policies, programs, and immediate responses during public health pandemics [14–16], very few countries, if any, had sufficient ethical guidance in place to afford them uncontroversial decisions during the outbreak of COVID-19 [17, 18].

The goal of this paper is to formulate an ethics and human rights criteria for evaluating public health measures and use it to reflect on the ethical propriety of those adopted by the government of Uganda to contain the spread of COVID-19. But before doing so, we first demonstrate the critical *importance* of ensuring that public health measures satisfy basic ethics and human rights criteria. Even though the ethical controversies that arise during PHEs pertain to what has been broadly dubbed the 3Rs – rationing of health resources; restrictions on individual liberties and freedoms; and responsibilities (of the various stakeholders) [19], this paper focusses on limitations of liberties and freedoms, and related burdens imposed on individuals and communities. It is hoped that this analysis will stimulate a long overdue public debate on ethical and human rights considerations in public health, including PHEs in Uganda, and potentially other Low and Middle Income Countries (LMICs). This hope echoes the WHO’s caution that prospective deliberations on ethical questions in PHEs is critical because, as the experience of the COVID-19 pandemic has demonstrated, the relevant ethical questions are particularly difficult to effectively address due to insufficient time once a pandemic has occurred [16].

To achieve the goal of this paper, we used content analysis to identify the measures adopted by the government of Uganda to contain the spread of COVID-19, the ethics and human rights considerations commonly recommended for public health responses and establish their importance. The results of our analysis indicate that during PHEs, it is generally ethically and legally acceptable for *some* of the individual liberties and freedoms to be suspended to meet public health goals. However, the study found that there are certain ethics and human rights considerations that should set boundaries for such limitations and derogations. In addition, we found that Uganda’s economy and health care system are very fragile in a manner that increases the population’s vulnerability to human rights violations and social injustice arising from very restrictive public health measures. We anticipate that since these social and economic features are not only associated with Uganda but also prevalent in most LMICs, our analysis is relevant to other similar contexts. With regard to ethical and human rights considerations in public health, our analysis found congruence among the various perspectives on basic ethics and human rights criteria for responses to public health threats. An assessment of some of the country’s initial responses to COVID-19 pandemic found that some of them are indefensible from an ethics and human rights point of view. In addition, feasible options would have satisfied a basic ethics and human rights criteria better, and would probably have achieved the public health goal in question. Below we begin by emphasizing the importance of integrating ethics and human rights considerations into the design and implementation of public health measures including during PHEs. Before evaluating Uganda’s responses to COVID-19 for their ethical propriety, we highlight the country’s relevant social and economic features as crucial circumstances needed to appreciate the analysis. Furthermore, we summarize some of the potentially controversial responses adopted and identify some of the basic ethics and human rights criteria for assessing them. Finally, we use these criteria to reflect on the possible ethical legitimacy of those measures, and offer some recommendations***.***

## Main body

### The case for ethics and human rights considerations in public health

The ethical principles such as reciprocity, transparency, non-discrimination, accountability, non-maleficence, equity, and others have been recommended to guide any implementation of restrictive and burdensome public health measures [19, 20]. It has also been observed that these ethical principles bear intrinsic value and are important in ensuring the effectiveness of the adopted measures [16, 20]. However, in designing and implementing public health measures including during PHEs such as the COVID-19 pandemic, there is a likelihood of regarding ethics and human rights considerations as of secondary importance. This is more probable in severely resource-limited settings like Uganda and other similar contexts in LMICs. The reasons are evident: since there is usually no sufficient time and resources to facilitate careful ethical deliberations in these circumstances [16], focus should exclusively be on implementing measures with prima facie potential effectiveness. This is what some claims in the media in Uganda have revealed in response to some of the potentially morally controversial measures adopted by the Government to contain the spread of COVID-19 [21]. However, the measures’ *inherent* potential to achieve a public health goal, and the extent to which such measures satisfy basic ethics and human rights criteria, play *complementary* roles in ensuring uptake and actual effectiveness of the adopted measures [22–26, 20]. Therefore, to contain pandemics such as COVID-19, ethical assessment of contemplated measures and their mode of implementation are as critical as their prima facie potential for effectiveness [27, 16, 20].

The assumption of the assertion of *complementarity* is that many public health measures adopted to reduce, and eventually stop the spread of human-to-human infections, largely depend on voluntary compliance by the public, and the simplicity of their enforcement. These two factors depend on the ethical legitimacy of such measures, which in turn depends on the extent to which those measures satisfy certain ethics and human rights criteria. A careful look at such criteria intuitively reveals that ‘ethically legitimate public health measures are easier to voluntarily comply with and/or enforce’. Consequently, it has been advised that when alternative potentially effective measures are identified, the principles of ethics and human rights should be applied to hone them and make them just, fair, non-discriminatory and *acceptable* [28] (emphasis added). We take as axiomatic a contention that it is this inherent *acceptability* of measures which is crucial for inducing voluntary compliance and facilitating their enforcement. However, the concept of *acceptability* itself presupposes a number of other specific ethical criteria, which lead to the public’s perception of the measures’ legitimacy. Some of such criteria can be seen in a recommendation in reference to the COVID-19 pandemic, that containment, mitigation, and suppression plans must be as *inclusive* and *equitable* as possible, or else they risk undermining response efforts [27].

The case for explicitly integrating ethical considerations in public health policy and program evaluation has been articulated as a complement to traditional ‘evidence’. The motivating concern for this view is that the traditional concept of ‘evidence’ exclusively focuses on the potential effectiveness of alternative policy measures without reflecting on how the ensuing actions will impact ethical-related goals of public health. Hence, this position is based on the need to capture some of the common but mostly implicit ethical goals of public health – ‘doing good’, ‘avoidance of harm’, ‘preventing or reducing avoidable health disparities (health equity), among others. This suggests a need for going beyond the traditional and mechanistic approach to health policy evaluation that relies on ‘evidence’ per se, to a more holistic one that captures the ethical-related goals of public health [20].

It is important to appreciate that in uncertain situations where there are overwhelming burdens on health systems such as those presented by the COVID-19 pandemic, it is extremely difficult to implement public health measures that are free of ethical controversy [18]. This is even more difficult in severely resource-limited countries like Uganda. This is so because, as it has been cautioned in reference to responses to the H1N1 influenza pandemic, in similar circumstances, minimalist measures are likely to be ineffective, while maximalist, disproportionate ones pose potential long-lasting negative effects on community trust, public services, social order, and the economy [29]. Generally, ethical controversies about public health measures can result from perceived deception in the form of deliberate under-reporting of statistics of the pandemic [30] or exaggeration of the same statistics; compulsory institutional quarantines at one’s own cost [7–9], or judicial detention of potentially infectious patients who are uncooperative [31].

It should be noted that some ethically controversial measures usually come with seemingly robust pragmatic justifications. However, their failure to satisfy ethics and human rights criteria will jeopardize their effectiveness. For example, deception in the form of deliberate under-reporting of the magnitude of the pandemic may be justified by the goal of staving off the devastating psychological impact of truthful reporting on the economy. On the other hand, such deception will lead to false low-risk perceptions among the public, which directly compromise public’s voluntary compliance with highly restrictive safety measures or complicate their enforcement. Such measures will be wrongly perceived as disproportionate, unnecessary and unreasonable in the circumstances; therefore, they will increase the spread of the infection. The reverse is true for deception in the form of exaggeration of the statistics – unnecessary speculations may devastate the economy and lead to the adoption of highly restrictive measures, thus *unnecessarily* limiting and derogating human rights. Furthermore, it is natural that perceptions of discrimination in the form of privilege-like exemptions for some people from compliance with highly burdensome measures such as institutional quarantine *– inequitable imposition of burdens* – will generally weaken a sense of obligation for voluntary compliance among the public and even make enforcement largely unsuccessful, or unnecessarily violate people’s rights.

The emerging insight is that the importance of explicitly integrating ethics and human rights considerations into the choice of effective policies and measures cannot be overstated. Our contention is that public health policies and measures chosen following a more holistic approach that combines ‘evidence’ and ‘ethics and human rights considerations’ as its criteria has better chances of success than a mechanistic one which relies on ‘evidence’ alone. Hence, if ‘evidence’ is the only input for such decisions, then there is a strong case for revisiting the traditional concept of ‘evidence’ as it applies to public health, to include the potential ethical and human rights impact of alternative policies, programs and measures.

### Uganda at the beginning of COVID-19 pandemic

In bioethics, it is generally agreed that social, economic and other circumstances are key to the appreciation of ethical evaluation of human choices and actions [32]. Therefore, before engaging in an assessment of the ethical propriety of some of the measures adopted by the government of Uganda against COVID-19, it is important to first highlight some of these circumstances to guide such reflection. The assumption here is that social and economic vulnerabilities including poverty, income inequalities and under-resourced health systems’ capacities increase susceptibility to human rights violations or failures and many ethical values. Estimated at 45.7 million people [33], about 34.6% of Uganda’s population live on $1.90 or less per day [34]. Being the eleventh poorest country in the world, Uganda’s Gross National Income (GNI) per capita is USD 620 [35], while the annual health expenditure per capita is about USD 36.71 [36]. In the Financial Year 2019/2020, Uganda allocated a mere 8.9% of the annual budget to health [37], which constituted approximately 9% of the Total Health Expenditure (THE). The external sources (donor and development aid for health) contributed 44%, while private health expenditure contributed about 47% [38]. In addition, due to high levels of unemployment (approximately 10%) [39], between 100,000 and 150,000 Ugandans were employed in non-professional occupations in the Middle East alone, mainly as domestic workers and casual laborers, with some earning as low as USD 250 per month [40]. To pre-empt our discussion, it is important to note that some of these people were returning home at the outbreak of COVID-19 and had to pay exorbitant quarantine fees. It is equally important to note that Uganda is no stranger to outbreaks of highly deadly infectious diseases, especially hemorrhagic fevers such as Ebola, Marburg and Congo Hemorrhagic fever for which credit has been given for effective response [41]. Despite this experience of infectious diseases, not much ethical analysis, if any, has been conducted on the public health measures adopted to contain the spread of previous epidemics in Uganda despite the WHO’s call on countries to do so. However, the country’s response to the COVID-19 pandemic has made such analysis an immediate necessity.

### Some of Uganda’s initial measures against COVID-19

In the wake of the COVID-19 outbreak, the government of Uganda adopted a number of public health measures with great potential of effectively containing the importation of the first cases and later localized widespread infection [7–9]. As mentioned earlier, Uganda’s precarious social and economic circumstances increase its population’s vulnerability to human rights violations or failures because of highly restrictive and burdensome public health measures. Even though many measures were implemented, only four will be analyzed to illustrate the argument of this paper.

#### Denying Ugandan citizens from the epicenter to return home

The government of Uganda’s first potentially controversial response to the threat of the spread of COVID-19 was a categorical refusal to return her citizens, particularly students studying in Wuhan, China, who had requested the government to evacuate them. The refusal was maintained despite a week-long public campaign to have the students returned from China [42]. One of the reasons in support of this measure was that, given the capacity of China’s health system compared to Uganda’s, the students were better off at the epicenter of the outbreak. The second reason was a worry about the risk of a possible importation of COVID-19 into Uganda’s fragile health system [8]. However, the government sent relief funds to the affected students to help them cope with the situation.

#### Implementation mode for mandatory institutional quarantine

The second controversial response was *the mode of implementing* mandatory institutional quarantine. In principle, the measure of a 14-day mandatory institutional quarantine applied to all travelers arriving from countries categorized as “Category 1”.^1^ This measure was initially implemented at the cost of those quarantined in privately owned facilities (hotels) at a cost of USD 100 per day, full board. This rate applied to everybody irrespective of his or her social and economic status. However, due to public outcry regarding the unaffordable costs, and the surging numbers of people who had to be quarantined, alternative facilities were designated at slightly more than half the original cost [43–46]. Eventually the Government agreed to cover the entire cost of the quarantine. For the purpose of this analysis, emphasis is on the originally preferred mode of implementing the quarantine.

#### Physical and social distancing

Another measure adopted by the government to contain the spread of COVID-19 was physical and social distancing. To implement this measure, the government suspended public transport and later all private transport; halted all gatherings of social, cultural, political, recreational, religious and other nature; and required people to ‘stay home’. The use of private transport would be allowed only with special permission from government authorities, specifically Resident District Commissioners (RDCs). Going by Uganda’s latest population and housing census, on average one RDC serves a population of between 500,000 in rural settings and about 2 million in the urban districts. To preempt the discussion below, it is important to add that the suspension of private transport was a blanket restriction except for health workers and those involved in COVID-19 response-related work. No exception was granted for patients including those with urgent health needs such as on-going medical treatments, expectant mothers, and people with other health emergencies. However, the government promised to provide alternative transport, managed by RDCs, to be used in health emergencies. However, it is important to note that access to the RDCs and the emergency means of transport was limited given the large size of districts both geographically and in terms of population sizes. It should also be noted that it was moreover difficult to travel to seek permission to use private vehicles or access the promised transport, especially if the distance to district offices were long enough to require traveling by vehicles.

#### Government response to violations of measures

According to the Uganda Penal Code Act, 1950 (Section 171), “Any person who unlawfully or negligently does any act which is and which he or she knows or has reason to believe to be likely to spread the infection of any disease dangerous to life commits an offence and is liable to imprisonment for seven years” [47]. The country’s Public Health Act of 1935 lists more penalties for culpable non-compliance with public health precautions [12]. In the wake of mandatory institutional quarantine, both the media and Government authorities reported non-compliance with this measure amidst allegations of bribery of some of the quarantine officials [48]. However, Government enforcement of the relevant laws differed, depending, *seemingly* on the social-class of culprits [44]. Responses to violations have included the following: confiscating of private vehicles of individuals who violated the suspension of both private and public transport measure; arresting and detaining persons that violated the curfew; and verbal reprimands, among others. The ethical controversy over the Government responses to violations is particularly about what *seemed* like a social-class-based response. These responses included immediate arrest and detention with a possible sentence of 7 years imprisonment for ordinary individuals who violated the quarantine [49], in contrast to mere verbal reprimand for Ministers, Members of Parliament and their dependents who equally violated the same measure [44].

In order to reflect effectively on the ethical propriety of the above measures, there is need to identify ethics and human rights criteria to guide the reflection. To devise basic criteria for this purpose, we reviewed some of the ethical and human right standards in public health generally and PHEs. The findings are summarized below.

### Ethics and human rights considerations in public health

Generally, the central ethical dilemma in public health is to balance respect for individual freedoms and liberties with the responsibility of governments to provide their citizens with sufficient protection in relation to health [6, 50, 24, 28, 51, 52, 16]. To guide this balancing act, scholarly suggestions and official guidance have been offered, from which this paper identifies some of the basic ethics and human rights criteria for assessing public health measures, including responses during PHEs [53, 16, 50, 11, 54, 55, 14, 15].

In his discussion on limits of individual liberty, John Stuart Mill offered, as a general criterion, what is now popularly known as the ‘Harm Principle’. It states, “The only purpose for which power can be rightfully exercised over any member of a civilized community, against his will, is to prevent harm to others” [10]. When applied to the public health discourse, this principle is used to justify the implementation of autonomy-limiting public health measures, especially if there is evidence that unconstrained exercise of certain individual freedoms and liberties – such as movement, association, privacy, among others, will lead to widespread infections or injuries to the public. On the basis of similar reasoning, the Siracusa Principles allow national governments to limit and derogate some human rights in certain situations, including public health emergencies (Clause 25) [11]. Consequently, the moral issue is not whether individual liberties and freedoms can be limited and derogated to achieve public health goals, but whether such burdens meet certain basic ethics criteria.

Nancy Kass’ “*An ethics framework for public health”* [53] provides a significant framework to guide the integration of ethics considerations in the design and implementation of public health programs. According to this framework, the analysis in the process of choosing appropriate public health policies, programs and measures it is important, primarily, to identify the policy or measures’ goals to be achieved. After listing alternative policies, programs or measures, it is important to evaluate each of them for their potential efficacy in achieving the target goal(s). In addition, it is important to estimate the burdens each of the measures will impose on the public, and then find the means of mitigating such burdens in the course of implementing the chosen measures. In addition, in case certain public health policies, programs or measures are judged burdensome and restrictive, Kass recommends that efforts should be made to identify alternative measures, which are equally effective but less burdensome. Further, since it is very difficult to entirely eliminate burdens from public health measures, especially those adopted during PHEs, justice demands that these burdens be equitably distributed among the population, as opposed to being shouldered by a few. Finally, effort should be made to ensure a fair balance between the benefits and burdens of the adopted public health programs or measures [53]. The ethical insights in these questions have been reflected in several related scholarly views [24, 28, 51, 52, 56, 29, 57, 19].

Furthermore, learning from the experience of the ethical gaps in response to previous pandemics, the WHO developed a set of ethical considerations to guide the development of public health responses to future influenza pandemics [16]. Even though these guidelines are intended to be used in preemptive public ethical deliberations, they still provide insights into the manner of managing ethical issues that arise during PHEs. Key considerations in these guidelines pertain to *balancing rights, interests and values* of societies, communities and individuals, and the clear definition of obligations of all categories of stakeholders [16] (emphasis added). This balancing act can be facilitated by referring to ethical principles. In the field of bioethics, the traditional ethical principles have been those proposed by Tom Beauchamp and James Childress – Respect for autonomy, Beneficence (doing ‘good’), Justice and Non-maleficence (avoidance of harm) [58]. Even though these principles have been largely applied in clinical medicine and health research, they have been said to be key principles in public health as well [20]. Other official ethical guidelines for designing and implementing public health measures have in several ways reiterated similar criteria [50, 18, 14, 15].

Of special interest are the *Siracusa Principles on the Limitation and Derogation Provisions in the International Covenant on Civil and Political Rights* [11] and General Comment No. 14 on Article 12 of the *Covenant on Economic Social and Cultural Rights* [55]. Clause 25 of the Siracusa Principles states, “Public health may be invoked as a ground for limiting certain rights in order to allow a state to take measures dealing with a serious threat to the health of the population or individual members of the population.” However, other clauses demand that state authorities do not act arbitrarily to unnecessarily violate human rights or impose *unreasonable* or *extremely burdensome* measures, which may not be *strictly required* to achieve public health goals in the prevailing circumstances. For example, “The severity, duration, and geographic scope of any derogation measure shall be such only as are strictly necessary to deal with the threat to the life of the nation and are proportionate to its nature and extent” [11]. Furthermore, “Whenever a limitation is required in the terms of the Covenant, to be “necessary,” this term implies that the limitation: (a) is based on one of the grounds justifying limitations recognized by the relevant article of the Covenant; (b) responds to a pressing public or social need; (c) pursues a legitimate aim; and (d) is proportionate to that aim” [11]. Similar constraints and the burden of proof being placed on governments are found in paragraphs 28 and 29 of *CESCR General Comment No. 14: The Right to the Highest Attainable Standard of Health (Art. 12****)*** [55].

From the above ethics and human rights recommendations, we can identify at least six ethical criteria for evaluating public health programs and responses to PHEs. This is not intended to be a complete set of ethics and human rights criteria for evaluating public health policies, programs and responses, but it is simply intended to be used to demonstrate the process of explicitly integrating ethics and human rights considerations in the design and implementation of public health interventions, including during PHEs.

#### The criteria

1. *Effectiveness*: Since it is a government obligation to protect the health of its population [59], the adopted measures must possess the potential to achieve the public health goal in question. In this case the goal is containing the spread of COVID-19.
2. *Strict necessity of the measures*: This criterion requires that if the adopted measures are very restrictive and burdensome to individuals and communities, there should be evidence that such measures are *required* to achieve the public health goal in question. In this case, being r*equired* means that it is *impossible* to achieve the target goal without such measures.
3. *Proportionality of the measures to the threat*: This requires that minimal public health threats should not lead to imposition of extremely burdensome and highly restrictive measures, but those that are *just enough* to meet the public health goal in question.
4. *Reasonability of the measures*: Several context-specific factors such as economic affordability by the public to comply with the measures, long-term impact of the measures on the lives of individuals and communities, economically, socially, psychologically among others, combine to determine the reasonableness of the adopted measures. Reasonableness would also involve the extent to which the adopted measures affect other competing public health goals and interests.
5. *Being the least restrictive measures*: This criterion is based on the assumption that usually, in every single public health situation, there are alternative routes to the achievement of a public health goal, which impose unequal burdens on individuals. Therefore, it is morally preferable that the least restrictive or burdensome of these measures be implemented to minimize the burden or mitigate harms on individuals.
6. *Equitable burden-distribution*: In bioethics generally, justice demands that burdens and opportunities for good health should be proportionately distributed to all concerned, and observe the principle of nondiscrimination.

These criteria are a rough summary of the various ethical and human rights considerations for public health programs, policies and measures as reviewed above. Even though this may not be a perfect summary, we hope that if adhered to it will go a long way towards addressing most of the common ethics and human rights concerns about public health measures, more so those adopted during PHEs.

## Discussion

In our analysis above we have emphasized the fact that it is important to integrate ethics and human rights considerations in the design and implementation of public health measures and responses, including during PHEs. We have also gone ahead to identify basic criteria to guide such a process. In addition, we have provided a summary of the social and economic circumstances in Uganda, which, we contend, are important for the appreciation of the ethical assessment of the public health measures we have identified. Using the criteria identified for this purpose, we now turn to the evaluation of the four public health measures we have chosen.

### Quarantine

Mandatory quarantine is generally a globally morally accepted public health measure; it is provided for in Uganda’s Public Health Act [12]. However, what is apparently contentious in this case is the mode of its implementation. Given the severe scarcity of health resources in Uganda, it could be argued that the Government genuinely lacked resources for quarantine services; and, therefore, to require every quarantined individual meet their costs was a pragmatically *necessary* compromise. However, even if this were the case, sympathy for this mode of implementing quarantine would still wane because of the initial exorbitant cost of USD 100 a day per person. To gauge the reasonableness of this measure, it is important to consider that normally, the government of Uganda pays a lump sum of approximately USD 33 to its mid-level civil servants for an overnight stay in the field within the country. Therefore, the basis used by the government to charge USD 100 per day for her ordinary citizens was not clear. In our opinion, this mode of implementing the quarantine was neither reasonable, necessary nor fair given Uganda’s social and economic conditions. Our moral concern is about the financial consequences and devastation of livelihoods for those quarantined using this mode. This concern is further supported by evidence that “Even short-term restrictions on freedom of movement can have significant — and possibly devastating —financial and social consequences for individuals, their families, and their communities” [15].

The other questions to reflect upon are whether this manner of implementing the quarantine was equitable and the least restrictive of the potential alternatives or not; and if there could have been feasible, less restrictive, and equally effective means to implement it. We mentioned earlier that even though the Government later agreed to cover the full cost of the quarantine, in order to illustrate the importance of ethical considerations, we will focus on the initially preferred mode of implementing this measure. For example, to estimate the magnitude of the burden and the impact of this measure as implemented, one needs to consider the burden it imposed on the Middle East returnees referred to above*.* For this category, it meant that each of the quarantined individuals needed approximately an entire one-half of their monthly salary to cover one night of the quarantine days. Furthermore, given Uganda’s GNI per capita (USD620), an average Ugandan would have needed an equivalent of more than two years of their gross incomes to cover 14 days of quarantine. Therefore, the burden would be even worse and disproportionate for the lowest quartile populations given the high income inequalities in the country (GINI Index of 42.8% in 2016) [60]. This would lead to rampant attempts at evading the quarantine. Regarding the possibility of alternative measures, as it turned out later, there were feasible and equally effective alternative but less burdensome. Later on, the costs of the quarantine were revised downwards and much cheaper quarantine facilities were provided; while eventually, the government agreed to foot the entire bill. This latter decision was a more equitable mode of implementing the quarantine. Consequently, the alleviation of the burden of quarantine fees increased voluntary compliance with this measure.

### Refusal to repatriate students

With regard to this measure, we admit that it is difficult to assert any straightforward position on its ethical legitimacy, especially in view of the gesture by the Government to send relief funds to the affected students. The issue here is not whether, by virtue of resource constraints, it was impossible to evacuate the students since the relief funds sent to them were sufficient to bring them back. Hence, the relevant question is whether it was fair to refuse to repatriate them for precautionary reasons, while at the same time allowing other travelers from Category 1 countries into the country. We observe that compared to travelers from other Category 1 countries, travelers from Wuhan had a much higher risk of being infected and this might *explain* this differential treatment. However, this explanation may not pass as a *justification* for this measure because the country had the capacity to quarantine, isolate and test the returning students as it did for other risky and actually infected travelers. The assumption of this concern is that it matters both morally and practically for individuals to be in their home country, which in this case was moreover by far safer. Further questions to reflect upon include, for example, whether there was a *least restrictive alternative* measure to prevent the spread of the virus into the country; whether this burden was *equitably* imposed on all citizens that desired to return to their country before total closure of borders, and whether such refusal was *strictly necessary* to meet the target goal. In our opinion, since Government had the capacity to repatriate, quarantine, isolate, test and manage cases of infected persons, this measure was *not strictly necessary*. Moreover, its impact was inequitably imposed on travelers in similar situations.

### Blanket suspension of private transport

Arguably, the justification of this restriction is compelling. Some owners of private vehicles took an unfair advantage of prohibition against public transport and used their vehicles to transport passengers at exorbitant fares, thus failing the crucial preventive measure of physical and social distancing. However, the controversy about this measure is the failure to grant exceptions, particularly for health-related emergencies, such as women in labor; diabetic patients; HIV-AIDS patients; sickle cell anemia patients; and patients with cardiac conditions among others. In all these cases, lapses of time are a critical factor for health outcomes. All these categories needed either special permission to travel to hospitals or to use transport promised by Government. Later on, it became extremely difficult to both access the promised transport and secure permission to use private transport. Echoing dissatisfaction with this measure, concern was raised that the permission procedure through the office of RDCs was not practical for pregnant mothers and persons with chronic diseases [61].

Another concern about the requirement to obtain special permission to travel is that patients had to justify their need to travel; therefore, this required them to reveal their confidential health information, including their HIV/AIDS status to be granted the permission, which was stigmatizing. Hence, they faced the dilemma between stigma which comes with disclosure of their health conditions to non-health workers and going without permission to travel and access essential health services. Using the above ethical criteria, the obvious questions would be whether denial of exceptions to such patients was a *strictly necessary* measure to achieve social distancing and what short and long-term impact this measure might have on concerned patients. In our considered opinion, the answer to these questions is in the negative because this mode of implementing social distancing was not strictly necessary since the number of those who would have benefited from the exception to this measure are a negligible proportion of the whole population. Therefore, failure to grant automatic exceptions to this measure for people with urgent health needs was unreasonable and the risks and burdens to the affected patient groups were disproportionate to the magnitude of the threat at hand.

### Social-class-based response to non-compliance

According to the WHO ethics guidance, “Restrictions on freedom of movement should be applied in the same manner to all persons posing a comparable public health risk” [15]. And, accordingly, membership in any disfavored or favored social group or class should not be used to determine on whom to impose restrictions. Reasons for imposing restrictions should be strictly related to the risks individuals may pose to others [15]. As mentioned earlier, one of the most controversial responses by government authorities is tolerating what *seems* like discrimination in the form of social-class-based response to violations of the measures relating to the quarantine. It is important to disclaim that there was no official position endorsing such unjustified preferential and highly risky treatment, nor was there any explicit official attempt to defend this inconsistency in the face of rampant public criticism. However, some socially high-ranking culprits received mere verbal reprimand while others were denied attendance of parliamentary sessions and cabinet meetings because of non-compliance with quarantine and the consequent risk to their high profile colleagues [44, 48]. This shows at least that the differential treatment was deliberate; and it is a case of discrimination and inequitable distribution of the burden of the adopted measures.

#### Effect of ethics and human rights failures on the implementation of measures

Earlier on, we contended that integrating ethical and human rights considerations in designing and implementing public health measures is partly important for improving their effectiveness. In other words, failure to adopt measures that satisfy basic ethics and human rights criteria has great potential of undermining the efforts. These theoretical contentions are corroborated by the findings of this study. Media analyses attributed maneuvers to evade quarantine to the exorbitant quarantine fees [45, 43, 49]. In this case, it can be assumed that those who offered bribes to evade the quarantine considered USD 200 of a bribe to be far cheaper (a lesser economic burden) than USD 1400, which according to some, was extortion [62]. In addition, during one of the media interviews about this problem, the Health Minister regretted the bad example set by high profile people refusing to be quarantined that made it extremely difficult to enforce the measure [48].

#### Final considerations and recommendations

The findings of our study and the recommendations we make below serve to strengthen the case for implementing the WHO advice to Member States to engage in prospective public deliberations on ethical considerations of the possible measures during public health pandemics. Our findings have revealed that even though governments may be willing to implement measures that meet ethics and human rights criteria, they may not be able and ready to do so unless the relevant deliberations are undertaken in advance. This is revealed by the government of Uganda’s continuous revision of some of the publicly criticized measures to make them less burdensome and hence *socially acceptable*. We take this as evidence for Government’s implicit acknowledgement of the importance of implementing ethically appropriate public health measures. We note that this paper does not have sufficient empirical data and space to make more specific recommendations on measures that should have been adopted and their modes of implementation. However, given the ethics and human rights criteria we have identified, the measures identified above should have been replaced by equally effective alternatives without the ethically problematic aspects that we have highlighted. We recommend that having learned from the COVID-19 experience, the government of Uganda and all LMICs in similar situations should gather more specific evidence in their contexts on how to effectively integrate ethics and human rights considerations into public health measures. This evidence should be used to develop locally-sensitive or responsive frameworks to guide explicit integration of ethics and human rights considerations in designing and implementing public health measures, including during PHEs. Secondly, given the magnitude and nature of the previous epidemics that informed existing WHO ethical guidelines compared to that of the current pandemic, it may be necessary for the WHO to go back to the drawing board, and integrate into such guidelines guidance that is more ethical based on lessons learned from COVID-19 pandemic.

## Conclusion

This paper intended to reflect on the ethical propriety of some of Uganda measures adopted to contain the spread of COVID-19. To strengthen the relevance of this work, we started with demonstrating the importance of integrating ethics and human rights considerations in designing and implementing public health measures, including during PHEs. The findings have revealed that the ethical legitimacy of public health measures is critical especially in ensuring their effectiveness, and such legitimacy depends on the extent to which those measures satisfy basic ethics and human rights criteria. Consequently, in designing and implementing public health measures, with or without PHEs, ethical and human rights concerns are a necessary complement to traditional evidence. Even though it is difficult to ascertain *moral culpability* arising from Governments’ initial responses to COVID-19, this potential exemption from strict moral culpability compensates neither for the negative impact of ethical gaps on the effectiveness of such measures nor for the long-term negative impact of such measures on the livelihoods of those who suffered extreme restrictive and burdensome measures. In addition, it has emerged that although some of the initially adopted measures somewhat fell short of the ethics and human rights criteria, the Government demonstrated willingness to improve the ethical status of such measures. Consequently, if the government of Uganda and others in comparable circumstances are to ensure ethical preparedness for future pandemics, it is very important that they strive to engage in prospective public deliberations on the ethical and human rights considerations in designing and implementing public health measures including during PHEs.

## Data Availability

Not Applicable.

## Abbreviations

COVID-19: Coronavirus Disease 2019
ESCR: Economic, Social and Cultural Rights
GNI: Gross National Income
LICs: Low Income Countries
LMICs: Low and Middle Income Countries
PHE: Public Health Emergency
PHEIC: Public Health emergency of International Concern
RDC: Resident District Commissioner
WHO: World Health Organization

## Footnotes

1 Category 1 countries are those where the prevalence COVID-19 was at such a level the risk of travelers from such Countries being infected with Virus was significant. They included China, Italy, UK, France, Spain and USA among others.
